# Mechanism of Action and Therapeutic Potential of Xanthohumol in Prevention of Selected Neurodegenerative Diseases

**DOI:** 10.3390/molecules30030694

**Published:** 2025-02-05

**Authors:** Anna Długosz, Błażej Błaszak, Damian Czarnecki, Joanna Szulc

**Affiliations:** 1 Faculty of Chemical Technology and Engineering, Department of Food Industry Technology and Engineering, Bydgoszcz University of Science and Technology, 85-326 Bydgoszcz, Poland; joanna.szulc@pbs.edu.pl; 2 Faculty of Health Sciences, Department of Preventive Nursing, Collegium Medicum in Bydgoszcz, Nicolaus Copernicus University in Torun, 85-821 Bydgoszcz, Poland; czarneckidamian@cm.umk.pl

**Keywords:** Alzheimer’s disease, amyotrophic lateral sclerosis, anti-inflammatory, antioxidant, neurodegenerative diseases, Parkinson’s disease, xanthohumol

## Abstract

Xanthohumol (XN), a bioactive plant flavonoid, is an antioxidant, and as such, it exhibits numerous beneficial properties, including anti-inflammatory, antimicrobial, and antioxidative effects. The main dietary source of XN is beer, where it is introduced through hops. Although the concentration of XN in beer is low, the large quantities of hop-related post-production waste present an opportunity to extract XN residues for technological or pharmaceutical purposes. The presented study focuses on the role of XN in the prevention of neurodegenerative diseases, analyzing its effect at a molecular level and including its signal transduction and metabolism. The paper brings up XN’s mechanism of action, potential effects, and experimental and clinical studies on Alzheimer’s disease (AD), Parkinson’s disease (PD), and amyotrophic lateral sclerosis (ALS). Additionally, challenges and future research directions on XN, including its bioavailability, safety, and tolerance, have been discussed.

## 1. Introduction

In recent years, neurodegenerative diseases have emerged as one of the most frequently discussed and analyzed health challenges. This increased attention is largely due to the aging global population and the corresponding rise in the prevalence of these conditions. It is estimated that 1% of individuals aged 60 years have Alzheimer’s disease (AD), with the prevalence rising to as high as 50% among those aged 90 years and older. For Parkinson’s disease (PD), approximately 0.15% of the general population is affected, while the prevalence increases to 1.5–2% among individuals aged over 70 years [1].

Neurodegenerative diseases are commonly classified based on their pathophysiological characteristics. They include conditions that impair cognitive functions and memory, as well as those that affect movement, communication, and respiratory abilities. Examples of neurodegenerative diseases include AD, PD, Huntington’s disease, amyotrophic lateral sclerosis (ALS), frontotemporal dementia, and spinocerebellar ataxias [2]. The progression of these diseases is influenced by various factors, including specific protein accumulations, anatomical vulnerabilities, and fundamental neuronal processes such as progressive neuronal dysfunction and death, oxidative stress, programmed cell death, neuroinflammation, proteotoxic stress, and abnormalities in the ubiquitin–proteasome and autophagosomal/lysosomal systems [3].

Oxidative stress is a common factor contributing to the progression of neurodegenerative diseases. This is attributable to the brain’s high oxygen consumption coupled with its relatively low antioxidant levels, making it particularly susceptible to oxidative damage. Oxidative stress occurs when the balance between pro-oxidants and antioxidants shifts in favor of pro-oxidants. Under normal physiological conditions, reactive oxygen species (ROS) (including O_2_, •OH, H_2_O_2_, and O_2_^−^•) play essential roles in cellular signaling pathways and transcription activation. However, when their levels exceed the cell’s antioxidant capacity, ROS can damage biological macromolecules, disrupt cellular functions, stimulate the production of pro-apoptotic proteins, and ultimately induce neuronal apoptosis [4,5].

**Figure 1:**
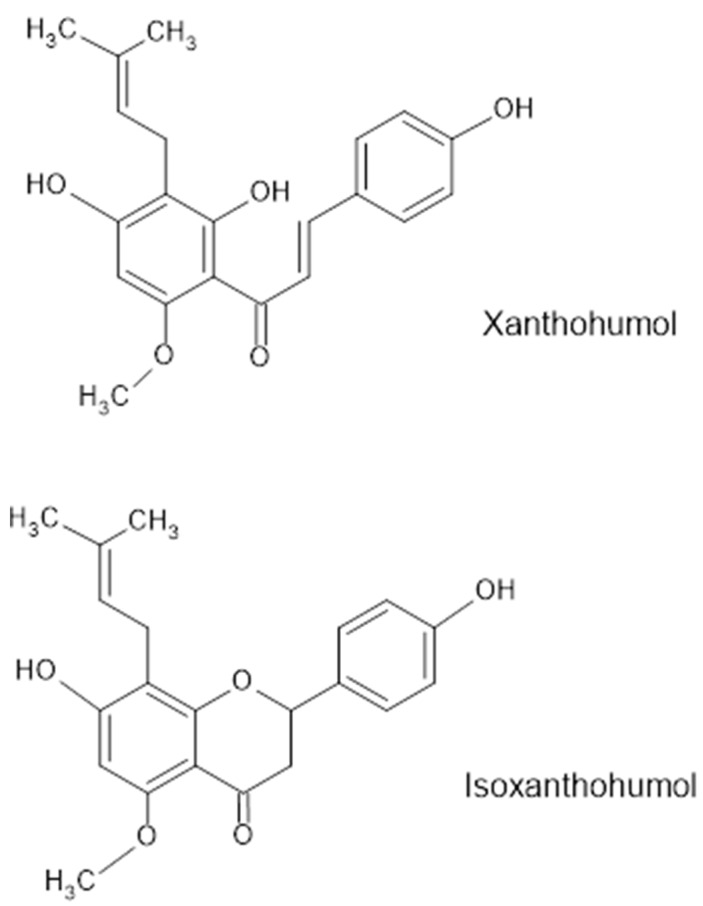
Structure of xanthohumol and isoxanthohumol [9].

Plants are a rich source of compounds that serve both nutritional purposes and act as specific plant responses to adverse environmental conditions or threats from pathogens (secondary metabolites). In medicine, plant secondary metabolites have found widespread applications due to their health-promoting properties, including anti-inflammatory, antimicrobial, antiaging, antioxidant, and anticancer activities [6]. Owing to the wide range of compounds and their impact on human health, both natural compounds and their chemically synthesized analogs are currently being investigated for their potential in preventing and treating various diseases, including neurodegenerative disorders. Confirmed activity in this regard has been demonstrated by isocumarins, which are derivatives of the widely distributed plant compound coumarin. These compounds, among other mechanisms, may positively influence the inhibition of disease progression by stimulating specific receptors [7,8]. Another compound with similar properties is xanthohumol (XN). XN (3′-[3,3-dimethylallyl]-2′,4′,4-trihydroxy-6′-methoxychalcone) is a natural chalcone belonging to the prenylflavonoid group, primarily found in the β-hard resin of female hop cones. In these cones, XN accounts for 0.1–1.0% of the dry mass [9,10]. Numerous derivatives of XN have been identified, with the basic compound structure and isoxanthohumol being the most well-known representatives (Figure 1).

The main dietary source of XN is beer, where it is introduced through hops. However, steps such as filtration, fermentation, and thermal treatment—during which isomerization of XN to isoxanthohumol occurs—result in a low final concentration of XN in beer. It is estimated that beer contains up to 0.69 mg/L of XN and as much as 3.44 mg/L of isoxanthohumol [9,11]. Despite this low concentration, the significant global production of beer and the large quantities of hop-related post-production waste present an opportunity to extract XN residues. This extraction could support the production of dietary supplements based on hop extracts. Consequently, research into XN has increasingly focused on methods for its efficient extraction [11].

**Figure 2:**
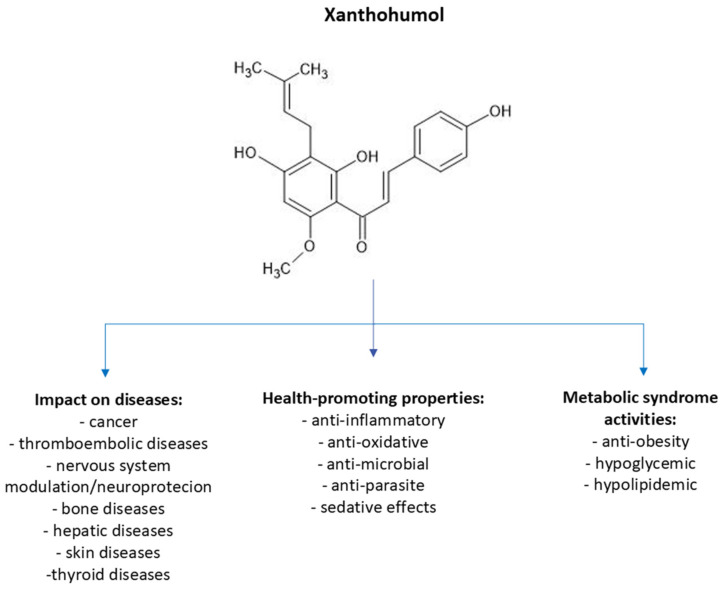
Pro-health properties of XN [9].

Numerous scientific studies, conducted both in vitro and in animal models, have attributed various health-promoting properties to XN (Figure 2). These include its positive effects on metabolic processes (e.g., in the context of overweight/obesity and glucose metabolism), anticancer properties through inhibition of procarcinogens, angiogenesis, and cancer cell growth—and even induction of apoptosis in cancer cells. Additionally, XN demonstrates antiatherosclerotic and neuroprotective effects [9,11,12,13]. Due to its beneficial health effects and the fact that it is easily available from plants and food industry byproducts, XN has the potential to be used in medicine, especially in neurodegenerative disease prevention. Thus, the therapeutic potential of XN requires a summary providing an insight into the research conducted to date, especially into its impact on biochemical mechanisms and expression of enzymes and proteins, which is the main objective of this paper.

## 2. Methods

We conducted an extensive literature search using the ISI Web of Science, Pub-Med, ScienceDirect, and Google Scholar databases to gather information on the mechanism of action and therapeutic potential of XN in the prevention of neurodegenerative diseases, including AD, PD, and ALS. The following keywords were used in the data search: (“xanthohumol” and “neurodegenerative diseases”); (“xanthohumol” and “antioxidant”); (“xanthohumol” and “anti-inflammatory”); (“xanthohumol” and “Parkinson’s disease”); (“xanthohumol” and “Alzheimer’s disease”); (“xanthohumol” and “amyotrophic lateral sclerosis”); (“neuroprotection” and “xanthohumol”); (“oxi-dative stress” and “neurodegenerative diseases”); (“neurodegenerative diseases” and “xanthohumol” and “oxidative stress”); (“inflammation” and “xanthohumol” and “neurodegenerative diseases”). There were no restrictions on data collection, and no language restrictions were applied during the search. We focused primarily on articles published in the last 15 years. After performing the initial search, we further examined the full text of identified studies to assess their eligibility for inclusion in this review. Editorials, conference abstracts, and studies with incomplete or unavailable data were excluded. Additionally, priority was given to studies that investigated the biochemical pathways involved in XN’s neuroprotective effects, specifically its antioxidant, anti-inflammatory, and anti-apoptotic properties. We included studies that provided evidence from both in vitro and in vivo experiments, as well as those exploring the efficacy of XN in preclinical models of PD, AD, and ALS. Meta-analyses, when available, were also considered to provide a quantitative overview of the available data.

## 3. Mechanism of Action of Xanthohumol

Xanthohumol has garnered significant attention in research due to its potent antioxidant properties. XN exhibits antioxidative effects primarily through its ability to scavenge ROS and RNS, thereby mitigating oxidative stress [14,15]. This free radical scavenging ability is largely attributed to the presence of hydroxyl groups in its structure [16], which can donate electrons to neutralize free radicals, thus preventing cellular damage. Additionally, XN influences intracellular signaling pathways, such as the Nrf2 pathway, which upregulates the expression of endogenous antioxidant enzymes like SOD, catalase, and glutathione peroxidase. Beyond direct radical scavenging, XN also modulates the redox state by chelating transition metals, thereby inhibiting metal-catalyzed oxidative reactions. These multifaceted mechanisms contribute to its protective role against oxidative damage, underpinning its potential therapeutic applications in managing diseases linked to oxidative stress, such as cancer, neurodegeneration, and cardiovascular disorders [14,17].

Chemical compounds with free radical scavenging activity typically neutralize reactive free radicals through several reaction mechanisms [18,19]. The primary mechanisms include HAT, which is characterized by a single-step process where the antioxidant, XN, donates a hydrogen atom to the free radical, neutralizing it. This results in the formation of a relatively stable antioxidant radical due to the delocalization of the unpaired electron [20]. Another mechanism is SET, where XN transfers a single electron to the free radical, neutralizing its unpaired electron and converting it into a more stable species. Additionally, a two-step mechanism called SPLET can occur, where the antioxidant first loses a proton, forming an anion, which then donates an electron to the free radical [20,21,22]. Tošovič et al. [23] investigated the antioxidative properties of XN, observing its behavior with selected free radicals and evaluating the effectiveness of various neutralizing mechanisms, including HAT, SET, and SPLET. Their study found that the HAT mechanism was most favorable for neutralizing CH_3_O• and HOO• radicals.

Different antioxidant mechanisms through which XN can react lead to varying activity and antioxidant power when laboratory methods are applied. These methods assess a substance’s ability to scavenge free radicals or inhibit oxidative processes [24]. Common assays include the DPPH assay, which measures the ability of antioxidants to donate electrons and reduce the DPPH radical to a colorless form [25]. The ABTS assay is another widely used method that detects the ability of antioxidants to scavenge the ABTS radical cation, resulting in a color change [26]. The FRAP assay, which evaluates the reduction of ferric ions (Fe^3^⁺) to ferrous ions (Fe^2^⁺), is also commonly used to assess antioxidant activity [27]. Additionally, the ORAC assay is a fluorescence-based method that measures the ability of a substance to quench free radicals in a controlled environment [28]. These laboratory methods, along with others such as the total phenolic content assay, help evaluate and compare the antioxidant efficacy of various substances, providing insight into their potential for preventing oxidative stress-related diseases.

The results obtained from antioxidant assays, such as DPPH, ABTS, FRAP, and ORAC, can be influenced by several factors that should be carefully controlled. Variability in results between different methods is affected by various conditions, including reaction time, pH, temperature, solvent type, matrix interference, XN extraction procedures, and the properties of the raw material (e.g., variety, cultivation location). Kontek et al. [29] pointed out that simple antiradical methods with artificial stable radicals, such as DPPH and ABST, have limitations when assessing the antioxidant potential of plant extracts or compounds, and it is not recommended to compare results evaluated with these tests to other antioxidant activity assays. Table 1 presents the antiradical activity of XN and hops-derived extracts using different antioxidant activity assays.

Antioxidant activity is commonly expressed in several units depending on the assay method used. The most frequently used units are TEAC, typically given in micromoles of Trolox equivalent per gram (µmol TE/g) or per milliliter (µmol TE/mL) of the sample. Another unit to describe antioxidant activity in Percent Inhibition or Percent Scavenging (%), which is a common unit used, is assays with DPPH or ABST as a free radical. FRAP is typically expressed in micromoles of Fe^2^⁺ per gram (µmol Fe^2^⁺/g) or per milliliter (µmol Fe^2^⁺/mL), but it can also be referred to as the Trolox equivalent. Similar to TEAC, ORAC values are often expressed in micromoles of Trolox equivalent per gram (µmol TE/g) or per milliliter (µmol TE/mL) of the sample. ORAC measures the ability of an antioxidant to quench peroxyl radicals in a controlled system. Antioxidant activity can also be presented as IC₅₀. IC_50_ refers to the concentration of the antioxidant required to scavenge 50% of the radicals or inhibit 50% of a specific oxidative process [19,20,21,22]. Such a multitude of methods and ways of expressing antioxidant activity makes quantitative comparison of this property difficult. Nevertheless, the results presented in Table 1 indicate that both pure XN (reagent), XN extracted from hops, and hops-derived extracts obtained by various methods have high antioxidant potential. Depending on the solvent used for extraction, hop extracts rich in XN were characterized by different antioxidant activity and evaluated using the same method [32]. The same situation is observed in the case of XN. It is worth mentioning that the XN SOAC value was about 14 times higher than that of vitamin E, recognized as one of the strongest antioxidants [35]. When compared to other water-soluble antioxidants, XN has a lower antioxidant capacity. For example, quercetin is characterized by an IC_50_ value that is 35 times lower than that of XN, at 0.0085 mg/mL and 0.294 mg/mL, respectively [29,39]. Similar findings can be observed in the case of ascorbic acid, a powerful antioxidant. XN and hops extracts exhibit antioxidant activity that is 91 to 375 times lower than that of ascorbic acid (Table 1). However, XN is a stronger antioxidant than isoxanthohumol or naringenin and is still considered a bioactive compound with significant potential in diseases triggered by oxidative stress.

The above-mentioned studies and their results demonstrate the antioxidative potential of XN and extracts rich in this compound. This creates many possibilities for application, especially in preventing oxidative stress-related diseases.

**Figure 3:**
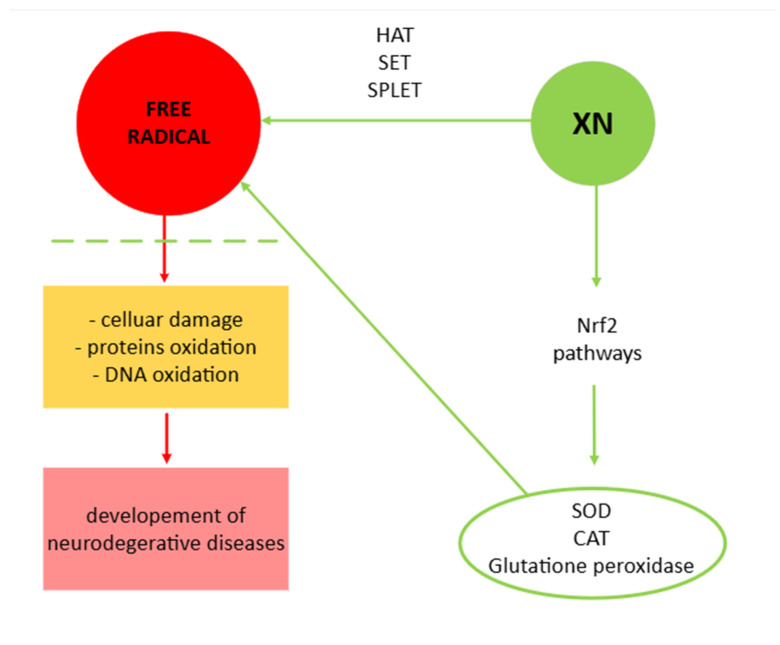
Mechanism of action of XN. Abbreviations: catalase (CAT); hydrogen atom transfer (HAT); nuclear respiratory factor 2 (Nrf2); sequential proton loss electron transfer (SPLET); single electron transfer (SET); superoxide dismutase (SOD).

The molecular mechanism of action of XN includes very complex pathways (Figure 3). XN reduces oxidative stress and regulates the activity of antioxidant enzymes [14,17,20]. XN is also a factor that impacts the cell cycle, e.g., inhibiting cell proliferation (particularly controlling cell cycle transcription during G1 and S phases in cancer cells) and inducing apoptosis (promoting cancerous cell death). XN is known for the inhibition of pro-inflammatory pathways. Blocking these pathways reduces the production of pro-inflammatory cytokines such as TNF-α and IL-6 [40] due to evidence that neurodegenerative diseases are related to the occurrence of oxidative stress, resulting in free radicals presence. More detailed mechanisms related to this group of diseases, especially AD, are presented in the following paragraphs.

## 4. Therapeutic Potential of Xanthohumol in Selected Neurodegenerative Diseases

### 4.1. Alzheimer’s Disease

Alzheimer’s disease is one of the most widespread neurodegenerative diseases, characterized by the accumulation of Aβ plaques and hyperphosphorylated tau protein in the brain, leading to the loss of cognitive functions. In recent years, research on XN has suggested that this natural flavonoid may possess neuroprotective properties that could counteract the pathological mechanisms of AD [41,42]. The hypothesis of mitochondrial cascade posits that damaged mitochondria increase the production of Aβ as a toxic oxidative stressor [43]. The free radicals damage DNA. Free radicals occur in histone-free mitochondrial DNA. There is a significant increase in double-strand breaks in the brain of people with AD, and dysfunctional DNA repair contributes to the progression [43].

In vitro studies have shown that XN exhibits strong antioxidant and anti-inflammatory properties. In cellular models, XN has been found to protect neuronal cells from oxidative stress, which is a key factor in the pathogenesis of AD [13,44,45]. One study conducted on human neuronal cells demonstrated that XN reduces the production of ROS, leading to decreased lipid peroxidation and cellular damage induced by Aβ [46,47,48]. Additionally, research has shown that XN inhibits the activity of the enzyme AChE, which may enhance cholinergic transmission, crucial for learning and memory processes [46,49].

In vivo studies on animal models, such as transgenic mice overexpressing amyloid, have also provided evidence of the therapeutic potential of XN. In one such study, long-term administration of XN to mice resulted in a significant reduction of Aβ deposits in the brain, which was correlated with improved performance in behavioral tests assessing cognitive functions, such as the Morris water maze test [42,50]. XN not only reduced Aβ accumulation but also lowered levels of inflammatory markers in the brain, such as IL-6 and TNF-α, suggesting its anti-inflammatory effects [51,52].

Additionally, XN is able to initiate the process of improving cognitive impairment by removing the inhibition of miRNA as miR-532-3p on Mpped1 in the mouse hippocampus [53]. However, it is miR-532-5p that has the most significant correlation with AD [54].

The molecular mechanisms by which XN may exert its protective effects in AD are diverse and complex [55,56,57]. One of the primary mechanisms is the modulation of oxidative stress, which plays a crucial role in the pathogenesis of AD. XN acts as a potent antioxidant, neutralizing ROS and reducing oxidative damage to DNA, proteins, and lipids. Additionally, XN may influence signaling pathways related to cellular stress responses, such as the Nrf2/ARE pathway (the nuclear factor E2-related factor 2—Nrf2; Nrf2-ARE pathway is an intrinsic mechanism of defense against oxidative stress), which regulates the expression of antioxidant and detoxifying enzymes. Activation of this pathway by XN can lead to enhanced protection of neurons against oxidative stress [58,59,60].

Another important mechanism is XN’s ability to inhibit neuroinflammation. Brain inflammation, triggered by the activation of microglia and astrocytes in response to Aβ accumulation, is one of the key processes contributing to the progression of AD. XN reduces the production of pro-inflammatory cytokines such as TNF-α, IL-1β, and IL-6 by inhibiting the activation of the NF-κB pathway, which is a major regulator of the inflammatory response [61].

Additionally, XN exhibits the ability to directly inhibit beta-amyloid aggregation. Studies suggest that XN may bind to Aβ monomers, stabilizing them and preventing their aggregation into neurotoxic oligomers and fibrils. This property could be particularly relevant in the context of AD prevention, where inhibiting the early stages of Aβ aggregation may slow the progression of the disease [41,62].

XN has yet to be extensively explored in the preclinical phases [63].

All these mechanisms suggest that XN may act in multiple ways, inhibiting key pathogenetic processes in AD, making it a promising candidate for further research on its potential use as a therapeutic agent in AD.

### 4.2. Parkinson’s Disease

Parkinson’s disease is a progressive neurodegenerative disorder primarily characterized by the loss of dopaminergic neurons in the substantia nigra, a critical area of the brain involved in motor control. This neurodegeneration leads to a decrease in dopamine levels, resulting in the hallmark motor symptoms of PD: tremors, bradykinesia (slowness of movement), rigidity, and postural instability [64]. The pathophysiology of PD also involves the formation of Lewy bodies, which are intracellular aggregates primarily composed of alpha-synuclein protein. The accumulation of these Lewy bodies is associated with further neuronal damage and contributes to the progression of the disease [65,66].

Accumulating evidence implicates oxidative stress as a key driver of the complex degenerating cascade underlying dopaminergic neurodegeneration in all forms of PD [67].

Additionally, inflammation is a factor contributing to the progressive neurodegenerative mechanism observed in PD [68].

In the context of PD, XN has been explored for its potential neuroprotective properties through a variety of experimental studies, including both cellular models and animal models. Cellular studies have demonstrated that XN exhibits potent antioxidant properties, which are crucial in countering oxidative stress associated with PD. For example, XN has been shown to mitigate the effects of oxidative stress in dopaminergic cell lines exposed to neurotoxins such as 6-OHDA and MPP+. These studies reveal that XN can reduce the production of ROS and protect against cellular damage induced by these toxins [69]. Studies using rodent models of Parkinson’s disease, such as those induced by 6-OHDA or MPTP, have shown that XN administration can significantly improve motor function and reduce neurodegeneration in the substantia nigra, a key area affected in PD. These findings suggest that XN exerts protective effects on dopaminergic neurons and mitigates the loss of dopaminergic terminals [70,71].

XN’s neuroprotective mechanisms in PD involve several key processes.

XN’s strong antioxidant properties are critical in neutralizing ROS and protecting dopaminergic neurons from oxidative damage. By scavenging free radicals and reducing oxidative stress, XN helps to preserve neuronal integrity and function [47,72].

XN has been shown to inhibit apoptotic pathways in dopaminergic neurons, reducing cell death that is commonly observed in PD. It achieves this by modulating key apoptotic signaling proteins, such as caspases, and by enhancing the expression of anti-apoptotic factors [55].

By reducing oxidative stress, XN helps to prevent damage to neuronal cells and tissues, contributing to overall neuroprotection. It modulates several oxidative stress-related pathways, including the Nrf2/ARE pathway, which enhances cellular defenses against oxidative damage [55].

XN’s anti-inflammatory effects are mediated through the inhibition of pro-inflammatory cytokines and the reduction of neuroinflammation. It influences the expression and activity of inflammatory mediators, such as TNF-α and IL-1β, thereby reducing neuroinflammatory responses in PD [73].

Preliminary clinical research into XN’s effects on PD is limited but promising. Recent studies are beginning to explore the potential therapeutic benefits of XN in human patients. Early-phase clinical trials are evaluating the safety and efficacy of XN in patients with Parkinson’s disease. These studies are focused on assessing XN’s ability to improve motor symptoms and quality of life in PD patients. Initial results indicate that XN is well-tolerated and may offer potential benefits in reducing disease severity and improving motor function [55,63,73]. These studies and findings collectively suggest that XN holds promise as a neuroprotective agent in PD through multiple mechanisms, including its antioxidant, antiapoptotic, and anti-inflammatory properties. Further research, especially larger clinical trials, will be essential to fully understand its therapeutic potential and efficacy in humans.

### 4.3. Amyotrophic Lateral Sclerosis

Amyotrophic lateral sclerosis is a progressive neurodegenerative disorder that primarily affects motor neurons, leading to muscle weakness, paralysis, and eventually respiratory failure. Current research into potential treatments for ALS has increasingly focused on natural compounds with neuroprotective properties, including XN [74,75,76].

Recent studies have explored the role of XN in treating ALS, particularly through preclinical research using animal models. These studies aim to understand how XN can influence the progression of ALS by protecting motor neurons from degeneration. In rodent models of ALS, such as those involving mutations in the SOD1 gene (a common genetic cause of ALS), XN has shown promise in enhancing motor neuron survival and delaying disease progression [55,77]. The SOD1 gene provides instructions for making an enzyme called superoxide dismutase, which is abundant in cells throughout the body. This enzyme attaches to molecules of zinc and copper to break down toxic, charged oxygen molecules called superoxide radicals. The molecules are byproducts of natural cell processes, and they should be broken down to avoid damaging cells [78]. For example, a study conducted on SOD1-G93A transgenic mice (a well-established ALS model) demonstrated that XN administration led to a significant increase in motor neuron survival and improved motor function. The study highlighted the compound’s ability to reduce oxidative stress markers and attenuate neuroinflammatory responses, which are critical factors in ALS pathology [79].

Additionally, emerging evidence from in vitro studies using motor neuron cell lines suggests that XN can modulate cellular stress responses, such as ER stress, and prevent apoptosis in motor neurons, further supporting its potential as a therapeutic agent in ALS [80].

Using XN as an Nrf2 modulator may have significant clinical potential in ALS therapy. The study suggests that natural polyphenols (e.g., XN) protect cortical cells against corticosterone-induced cytotoxicity and enhance cell survival via modulation of the Nrf2 pathway [81,82,83].

### 4.4. Potential Therapeutic Benefits in Selected Neurodegenerative Diseases

Oxidative stress plays a central role in ALS pathogenesis, contributing to the degeneration of motor neurons. XN’s potent antioxidant properties have been shown to neutralize ROS and reduce oxidative damage in neuronal cells. This reduction in oxidative stress helps to preserve motor neuron function and delay the onset of symptoms in ALS models [84,85].

Inflammation is another critical factor in ALS, with activated microglia and astrocytes releasing pro-inflammatory cytokines that exacerbate motor neuron damage. XN has demonstrated anti-inflammatory effects by downregulating the production of cytokines such as TNF-α and IL-1β, thereby mitigating the inflammatory responses in the CNS. This modulation of the immune response could slow the progression of ALS and improve patient outcomes [86,87].

XN’s neuroprotective effects extend to enhancing the survival and function of motor neurons. In experimental models, XN has been shown to protect against glutamate-induced excitotoxicity, a major driver of motor neuron death in ALS. By stabilizing cellular homeostasis and preventing excessive calcium influx, XN helps maintain motor neuron integrity and supports neuromuscular function [88,89]. XN is emerging as a promising candidate for ALS treatment due to its multifaceted neuroprotective properties. While current research is primarily preclinical, the findings provide a strong rationale for further investigation, including clinical trials to evaluate XN’s therapeutic potential in ALS patients. Future studies should focus on optimizing dosage and delivery methods and understanding the long-term effects of XN treatment in ALS.

Preclinical results indicate the high efficacy of XN in reducing neuronal damage and delaying the progression of neurodegenerative diseases in animals. For instance, a study conducted on a rat model of AD demonstrated that XN significantly improved cognitive function, as assessed by the Morris water maze test, which is commonly used to evaluate spatial learning and memory in animals. This study also reported a marked reduction in Aβ plaque accumulation, a hallmark of AD pathology [90]. Another example comes from research on PD, where XN administration in a mouse model led to a decrease in oxidative stress, as evidenced by lower levels of MDA and increased activity of antioxidant enzymes such as SOD and CAT [91,92]. These findings highlight the potential of XN in mitigating the oxidative damage associated with neurodegeneration.

Moreover, studies have shown that XN can modulate neuroinflammation, which plays a crucial role in the progression of neurodegenerative diseases. In a recent experiment involving a model of multiple sclerosis (EAE), XN treatment resulted in reduced expression of pro-inflammatory cytokines like TNF-α, IL-6, and IL-1β in brain tissue, alongside improvements in motor function [93,94]. This anti-inflammatory action underscores its potential as a therapeutic agent in diseases characterized by chronic inflammation. One study suggested that XN has the potential to develop into a therapeutic agent for improving glutamate-related nervous system diseases. This is important because toxicity induced by excessive glutamate has been implicated in many brain disorders [95].

XN exhibits neuroprotective properties and supports neuroregeneration, making it a promising compound for neurological health applications [96,97,98]. This process is important in the induction of neuronal differentiation for regenerative medical approaches based on multipotent NSCs. These cells are present in the adult human brain throughout life [99,100] and are capable of self-renewal and differentiation into cell types of the brain. Neural stem cells respond to external stimuli such as the learning process [100,101], physical activity [100,102], and molecules like XN [103]. XN, as a small molecule, may have the potential to induce the body’s own regenerative mechanisms and support healing after ischemic insults and other neurotoxic events [100].

The study suggests that oral ingestion of low doses of XN can be sufficient to alter lipoteichoic acid-dependent immune responses of monocytes in the peripheral blood of healthy humans. This outcome further showed that the beneficial effects of XN are related to a suppression of the CD14-/TLR2-dependent activation of cells [104].

The findings from these preclinical studies suggest that XN may play a key role in preventing neurodegenerative processes, particularly by reducing oxidative damage, inhibiting Aβ accumulation, and decreasing neuroinflammation. However, further research, including well-designed clinical trials, is necessary to confirm these effects in humans and determine the appropriate therapeutic dosing. Despite the promising animal data, human studies are still limited, and the pharmacokinetics of XN in the human brain remain to be fully elucidated.

## 5. Challenges and Future Research Directions on Xanthohumol

### 5.1. Issues Related to Bioavailability

One of the key challenges in harnessing the full therapeutic potential of XN is its limited bioavailability. Despite promising preclinical results demonstrating its wide range of biological activities—such as antioxidant, anti-inflammatory, anticancer, and antimicrobial properties—the translation of these findings into effective clinical applications is hindered by several pharmacokinetic limitations. Understanding and addressing these challenges is crucial for advancing XN-based therapies [46,105].

XN, like many hydrophobic polyphenols, exhibits poor water solubility, which significantly impairs its absorption in the gastrointestinal tract. This low solubility restricts the dissolution of the compound in the aqueous environment of the digestive system, leading to suboptimal absorption through passive diffusion. According to recent studies, enhancing the solubility of XN is a critical area for future research. Approaches such as the use of cyclodextrin complexes, micellar formulations, or nano-encapsulation have shown promise in improving the solubility and overall bioavailability of XN in experimental models [46,106,107].

Following ingestion, XN undergoes extensive biotransformation in the liver and intestines, which limits its presence in the bloodstream. Phase I and Phase II metabolic processes, including oxidation, reduction, and conjugation reactions (such as glucuronidation and sulfation), lead to the formation of various metabolites, including isoxanthohumol and 8-prenylnaringenin. These metabolites, while bioactive in some cases, may have different pharmacokinetic profiles and may not fully retain the biological potency of the parent compound. A study by Legette et al. [108] highlights that XN is rapidly converted to isoxanthohumol upon ingestion, which poses a challenge for achieving therapeutic concentrations of the parent compound in the systemic circulation. Future research should focus on strategies to modulate the metabolic pathways of XN to preserve its bioactivity. This could involve the development of prodrugs, enzyme inhibitors, or metabolic pathway modulation to reduce the rate of biotransformation [109,110,111].

In addition to rapid metabolism, XN has a relatively short half-life, which further reduces its therapeutic efficacy. Studies have indicated that the plasma half-life of XN is limited to a few hours post-ingestion, necessitating frequent dosing to maintain therapeutic levels in the body. However, such frequent dosing may not be feasible or desirable, particularly in chronic treatment settings. To address this issue, future research should investigate delivery systems that provide controlled or sustained release of XN, ensuring more stable plasma concentrations over an extended period. Nanotechnology-based delivery platforms, such as liposomes and polymeric nanoparticles, hold the potential for improving the pharmacokinetics of XN, enhancing both its bioavailability and half-life [112,113]. However, attempts are being made to develop formulas to increase the bioavailability of XN [114].

Nanoparticles, liposomes, and nanoemulsions have been explored as carriers to improve the absorption, stability, and distribution of XN. These systems can protect the compound from degradation in the digestive tract, facilitate its absorption, and extend its circulation time in the body [115,116,117]. Co-administration with absorption enhancers, such as piperine or other bioactive compounds, has been shown to improve the intestinal absorption of poorly soluble compounds like XN. This strategy could be investigated further to determine its applicability in enhancing XN bioavailability [118,119]. Cyclodextrin complexes have been reported to improve the solubility and stability of hydrophobic compounds. Cyclodextrin-based inclusion complexes with XN may improve its aqueous solubility, thus enhancing gastrointestinal absorption and systemic bioavailability [46,120]. Designing prodrugs that undergo enzymatic or chemical conversion to XN in the body can provide a controlled release mechanism that improves the compound’s stability and reduces its rapid metabolism [121].

### 5.2. Safety and Tolerance of Xanthohumol

**Figure 4:**
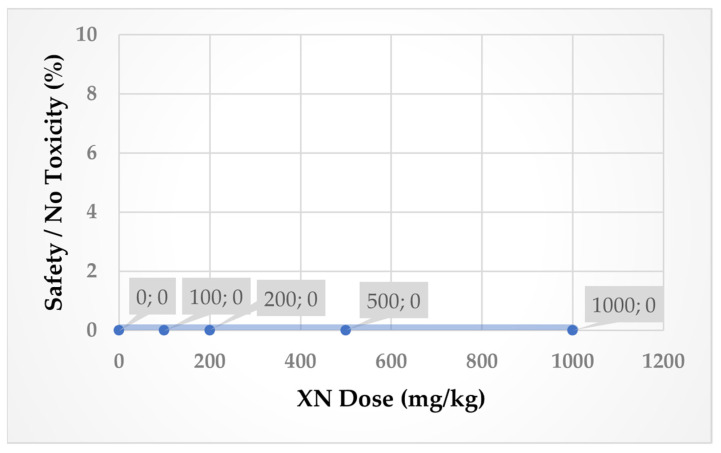
XN dose and safety/no toxicity in preclinical studies.

**Figure 5:**
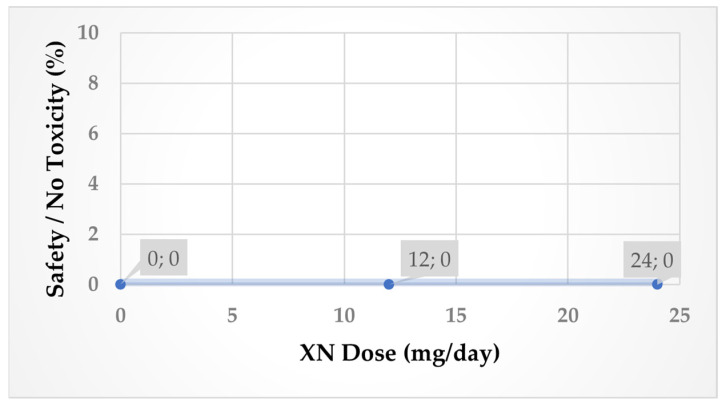
XN dose and safety/no toxicity in clinical studies.

Preclinical and clinical research indicates that XN, a prenylated flavonoid derived from hops, is generally well tolerated at moderate doses. In vitro and in vivo studies have not reported significant toxic effects at doses commonly tested for therapeutic purposes. For instance, rodent studies demonstrated safety at doses up to 1000 mg/kg, while human clinical trials observed no serious adverse events at doses between 12 mg and 24 mg daily [122]. To facilitate understanding, Figure 4 and Figure 5 illustrate the relationship between XN concentrations and observed effects, highlighting safe dosage ranges for both preclinical and clinical studies. The graphs delineate effective therapeutic doses (e.g., neuro-protection, metabolic benefits) and identify areas where safety data remain insufficient, such as chronic high-dose exposures.

However, while these findings are promising, the long-term safety of XN has not been thoroughly investigated. Most clinical trials have been limited to short durations, and the effects of chronic exposure remain uncertain. The potential for cumulative effects, particularly on liver and kidney function, as well as on metabolic pathways, warrants further investigation in both preclinical and clinical settings [108].

Although XN exhibits low toxicity at moderate doses, higher concentrations could potentially pose risks, as suggested by its structural similarity to other flavonoids, which can influence estrogenic and anti-estrogenic activity in some tissues [122]. Prolonged high-dose exposure might also interact with cytochrome P450 enzymes, affecting drug metabolism pathways and leading to potential drug interactions. This calls for further studies to determine the safety threshold and to explore any long-term metabolic or hormonal effects of XN supplementation.

Individuals with pre-existing liver conditions or those on medications metabolized by the liver should exercise caution with XN supplementation. Additionally, patients undergoing treatment for hormone-related cancers should consult a healthcare provider before using XN due to its potential to modulate estrogen pathways.

In conclusion, while current evidence supports the general safety of XN in moderate doses, long-term, and higher-dose studies are necessary to fully understand its safety profile and potential interactions, particularly for individuals using concurrent medications.

Authors of a study in mice models referred to equivalent doses in humans, stating that applications of XN can be expected to be safe in humans [123]. Human studies have confirmed the safety of XN. After standardizing an extract of spent hops to these marker compounds, an escalating dose study was carried out in menopausal women to evaluate safety and pharmacokinetics. There was no effect on selected hormones and blood clotting. The maximum serum concentrations of the prenylated phenols (including XN) reached 2 to 7 h, indicating slow absorption. Slow absorption and enterohepatic recirculation contributed to half-lives exceeding 20 h. The authors’ study confirms that short-term consumption of a standardized preparation of spent hops (containing, among others, XN) is safe for patients (women) and that once daily dosing might be appropriate [124].

Studies in the different areas of medicine (therapy for patients in intensive care), where XN was administered enterally three times a day every 8 h at a dose of 1.5 mg/kg body weight (4.5 mg/kg body weight/day) for 7 days [125], showed that treatment with XN improved the clinical course and lower the severity of the inflammatory response and mortality rate [126].

### 5.3. Future Research Directions

Despite the promising therapeutic potential of XN, significant gaps remain in the understanding of its pharmacokinetics, long-term safety, and clinical efficacy, particularly in treating complex diseases. Future research should prioritize improving XN’s bioavailability, exploring its potential in neurodegenerative diseases, and conducting more extensive long-term safety studies [122]. Only through these efforts can XN transition from promising preclinical findings to effective clinical therapies.

Recent research [127] in humans shows that 24 mg daily XN taken over an eight-week period was safe and well-tolerated by healthy adults and did not find abnormal values in clinical biomarkers, including those of renal and hepatic function, electrolytes, fasting glucose, and blood counts. It suggests safety and a lack of harm to major organs of the body. There were no severe or Food and Drug Administration-defined serious adverse events, but non-serious adverse events were documented in both the placebo and XN groups. Findings related to tolerability suggested that XN did not negatively influence quality of life.

XN continues to garner interest for its broad-spectrum biological activities, extending beyond its well-documented antioxidant and anti-inflammatory properties. Emerging research highlights its potential in the treatment of various diseases, including cancer, cardiovascular disorders, diabetes, and metabolic syndromes, as well as its neuroprotective effects.

XN’s potential as an anticancer agent has been extensively studied. Its chemopreventive effects are linked to its ability to induce apoptosis, inhibit cancer cell proliferation, and interfere with angiogenesis. XN has been shown to suppress the growth of various cancer cell lines, including breast, colon, prostate, and liver cancers, by modulating key signaling pathways such as NF-κB and PI3K/Akt/mTOR [122]. XN can interfere with the initiation, promotion, and progression phase of carcinogenesis by a variety of inhibitory mechanisms [128]. Furthermore, its ability to enhance the effectiveness of conventional chemotherapeutics and mitigate drug resistance positions XN as a promising adjunct in cancer treatment.

XN demonstrates cardioprotective effects, largely attributed to its antioxidant activity and ability to reduce inflammation. It has been shown to improve endothelial function, reduce oxidative stress, and inhibit the oxidation of LDL, a key factor in atherosclerosis development. Studies also suggest that XN can modulate lipid metabolism, reduce cholesterol levels, and improve overall cardiovascular risk profiles, making it a candidate for treating cardiovascular diseases [122].

In preclinical models, XN has exhibited the ability to improve insulin sensitivity, regulate blood glucose levels, and ameliorate lipid profiles, positioning it as a potential therapeutic for type 2 diabetes and metabolic syndrome. It influences several key metabolic pathways, including AMPK activation and PPARγ modulation, which are crucial for regulating glucose homeostasis and lipid metabolism. These findings suggest that XN may help prevent complications associated with diabetes, such as cardiovascular disease and liver dysfunction [129].

The neuroprotective properties of XN stem from its potent antioxidant, anti-inflammatory, and antiapoptotic effects. XN’s neuroprotective properties are driven by its antioxidant, anti-inflammatory, and antiapoptotic effects. It has demonstrated potential in protecting against neurodegenerative diseases such as AD and PD by reducing oxidative damage, inhibiting neuroinflammation, and preventing neuronal death. This makes XN a candidate for future therapies aimed at slowing the progression of these debilitating disorders, although more clinical research is required to confirm these effects in humans [41,130]. The neuroprotective effects of XN showed that XN is a promising supplement for ischemic stroke protection. The experimental studies showed that XN could revive neuronal apoptosis induced by oxygen–glucose deprivation by preventing oxidative stress injury (effects of the inhibition of phosphorylation of p38-MAPK and the mediation of nuclear Nrf2 activation) [47].

Due to its robust anti-inflammatory and antioxidant capabilities, XN may also have therapeutic applications in the treatment of metabolic and autoimmune diseases. Its ability to modulate immune responses, coupled with its effect on lipid metabolism, positions it as a potential candidate for managing conditions like rheumatoid arthritis and lupus. Research into its role in regulating immune cell activity, particularly in reducing pro-inflammatory cytokines, suggests that it could be a novel therapeutic approach for chronic inflammatory diseases [131,132].

Great hopes are placed on the lack of negative impact of XN on the microbiome and mental health [127].

The wide-ranging biological activities of XN make it a promising candidate for various therapeutic applications, from cancer and cardiovascular disease to diabetes and neurodegeneration. However, further clinical research is essential to fully validate its efficacy and safety in humans, as well as to optimize its bioavailability for therapeutic use.

## 6. Conclusions

Xanthohumol (XN), a bioactive flavonoid found in hops, has emerged as a promising candidate for the prevention and treatment of neurodegenerative diseases, thanks to its diverse mechanisms of action. The experimental evidence highlights XN’s potent antioxidant capabilities, which play a crucial role in mitigating oxidative stress—a key factor in the pathogenesis of conditions such as Alzheimer’s, Parkinson’s, and Huntington’s diseases. By scavenging free radicals and upregulating the body’s endogenous antioxidant defenses, XN protects neurons from oxidative damage. Notably, in amyotrophic lateral sclerosis (ALS), preclinical studies suggest that XN can help preserve motor neurons, even in the presence of SOD1 gene mutations.

Moreover, XN exhibits significant anti-inflammatory properties, which are particularly important given the central role of neuroinflammation in many neurodegenerative disorders. By inhibiting pro-inflammatory mediators such as NF-κB, TNF-α, and IL-1β, XN effectively reduces the chronic activation of microglia and astrocytes, thereby preventing further neuronal damage. This multifaceted anti-inflammatory action aligns with current trends emphasizing the importance of controlling inflammation to maintain neuronal health.

In addition, XN influences key neuroprotective pathways, notably the Nrf2/ARE pathway. Activation of Nrf2 leads to the expression of antioxidant and cytoprotective genes, enhancing neuronal resilience to various stressors. This is particularly relevant in Alzheimer’s disease, where the accumulation of amyloid-beta (Aβ) and hyperphosphorylated tau proteins contributes to cognitive decline. Experimental results indicate that XN reduces Aβ production and aggregation and inhibits tau hyperphosphorylation, thus potentially slowing the progression of the disease.

Another critical aspect of XN’s action is its role in supporting mitochondrial function, a vital factor in neurodegenerative diseases. By maintaining ATP production, reducing mitochondrial oxidative damage, and promoting mitochondrial biogenesis, XN helps sustain neuronal energy metabolism, which is crucial for cognitive function and overall brain health. This aligns with the current scientific focus on preserving mitochondrial health as a strategy to combat neurodegenerative disorders.

Finally, studies have demonstrated that XN positively impacts synaptic plasticity, enhances memory, and prevents cognitive impairment, which underscores its potential as a therapeutic agent. However, despite these promising findings, the bioavailability of XN in humans remains a challenge. Early clinical trials, such as those conducted in 2021, suggest that XN supplementation is safe and beneficial for gut microbiota and overall quality of life. Future research should aim to optimize delivery methods and dosages to fully realize XN’s therapeutic potential.

## Figures and Tables

**Table 1:** Antiradical activity of xanthohumol, hops-derived extracts, and selected compounds using a variety of antioxidant activity assays.

Sample	Type of Antiradical Activity Assay	Antioxidant Activity	Antioxidant Activity Compared to Ascorbic Acid (IC_50_sample/IC_50_AA)	References
Hops-derived hexane extract	ABTS	IC_50_ = 0.499 mg/mL	nd.	[29]
Hops-derived hexane extract	DPPH	IC_50_ = 0.294 mg/mL	91.9	[29]
Xanthohumol (reagent ≥ 99%)	ABTS	IC_50_ = 0.660 mg/mL	nd.	[29]
Xanthohumol (reagent ≥ 99%)	DPPH	IC_50_ > 1.2 mg/mL	>375	[29]
Hops-derived methanolic extract	ABTS	IC_50_ = 0.535 mg/mL	nd.	[29]
Hops-derived methanolic extract	DPPH	IC_50_ = 0.484 mg/mL	151	[29]
Xantohumol (reagent > 98%)	ORAC	23.447 mM TE/g	nd.	[30]
Xantohumol (reagent > 98%)	HORAC	72.245 mM TE/g	nd.	[30]
Xanthohumol-rich extract (Xantho-Flav, 90% XN)	ORAC	22.107 mM TE/g	nd.	[30]
Xanthohumol-rich extract (Xantho-Flav, 90% XN)	HORAC	55.662 mM TE/g	nd.	[30]
Hops-derived acetone extract	ABTS	0.31 mM TE/L	nd.	[31]
Hops-derived acetone extract	FRAP	0.27 mM TE/L	nd.	[31]
Hop leaves-derived methanolic extract (98% *v*/*v*, extraction time 60 min)	DPPH	3.72 mg TE/g	nd.	[32]
Hop leaves-derived methanolic extract (98% *v*/*v*, extraction time 60 min)	FRAP	8.55 mg TE/g	nd.	[32]
Hop leaves-derived ethanolic extract (70% *v*/*v*, extraction time 60 min)	DPPH	3.61 mg TE/g	nd.	[32]
Hop leaves-derived ethanolic extract (70% *v*/*v*, extraction time 60 min)	FRAP	6.18 mg TE/g	nd.	[32]
Hops-derived acetone extracts (90% *v*/*v*)	FRAP	63.5 ÷ 101.6 μM TE/g DW	nd.	[33]
Hops-derived acetone extracts (90% *v*/*v*)	ORAC	1069 to 1910 μM TE/g DW	nd.	[33]
Xanthohumol (reagent > 96%)	ORAC	24,745.1 μM TE/g DW	nd.	[23]
Xanthohumol (reagent > 96%)	FRAP	12,689.1 μM TE/g DW	nd.	[23]
Hops-derived extracts (different varieties)	DPPH	33.6 ÷ 77.7%	nd.	[34]
Xanthohumol	ORAC	4.2 TE	nd.	[35]
Xanthohumol	SOAC (vitamin E equivalent)	14.1	nd.	[35]
Quercetin	DPPH	IC_50_ = 0.0085 mg/mL	2.7	[35]
Resveratrol	DPPH	IC_50_ = 0.013 mg/mL	4.1	[36]
Butylated hydroxytoluene (BHT)	DPPH	IC_50_ = 0.0083 mg/mL	2.6	[37]
Isoxanthohumol	DPPH	IC_50_ = 2.971 mg/mL	928	[38]
Naringenin	DPPH	IC_50_ = 6.234 mg/mL	1948	[38]
Ascorbic acid	DPPH	IC_50_ = 0.0032 mg/mL	1.0	[38]

Abbreviations: 2,2′-azinobis(3-ethylbenzothiazoline-6-sulfonic acid) (ABTS); 2,2-diphenyl-1-picrylhydrazyl (DPPH); oxygen radical absorbance capacity (ORAC); hydroxyl radical antioxidant capacity (HORAC); ferric reducing antioxidant power (FRAP); singlet oxygen absorbance capacity (SOAC); half-maximal inhibitory concentration (IC_50_); Trolox equivalent antioxidant capacity (TE); dry weight (DW); half-maximal inhibitory concentration of Ascorbic Acid (IC_50_AA); no data (nd.).

## Data Availability

No new data were created.

## Abbreviations

1-methyl-4-phenyl-1,2,3,6-tetrahydropyridine	MPTP
1-methyl-4-phenylpyridinium	MPP+
2,2′-azinobis(3-ethylbenzothiazoline-6-sulfonic acid)	ABTS
2,2-diphenyl-1-picrylhydrazyl	DPPH
5′AMP-activated protein kinase	AMPK
6-hydroxydopamine	6-OHDA
acetylcholinesterase	AChE
adenosine triphosphate	ATP
Alzheimer’s disease	AD
amyotrophic lateral sclerosis	ALS
antioxidant response element	ARE
beta-amyloid	Aβ
catalase	CAT
central nervous system	CNS
cluster of differentiation 14	CD14
endoplasmic reticulum	ER
experimental autoimmune encephalomyelitis	EAE
ferric reducing antioxidant power	FRAP
half-maximal inhibitory concentration	IC_50_
hydrogen atom transfer	HAT
hydrogen peroxide	H_2_O_2_
hydroperoxy radical	HOO•
hydroxyl radical	•OH
hydroxyl radical antioxidant capacity	HORAC
interleukin-1β	IL-1β
interleukin-6	IL-6
low-density lipoprotein	LDL
malondialdehyde	MDA
mammalian target of rapamycin	mTOR
metallophosphoesterase domain containing 1	Mpped1
methoxy radical	CH_3_O•
neuronal stem cells	NSCs
nuclear factor-κB	NF-κB
nuclear respiratory factor 2	Nrf2
oxygen radical absorbance capacity	ORAC
p38 mitogen-activated protein kinases	p38-MAPK
Parkinson’s disease	PD
peroxisome proliferator-activated receptor gamma	PPARγ
phosphoinositide 3-kinase	PI3K
protein kinase B	Akt
reactive nitrogen species	RNS
reactive oxygen species	ROS
sequential proton loss electron transfer	SPLET
single electron transfer	SET
singlet oxygen absorbance capacity	SOAC
superoxide anion	O_2_•^−^
superoxide dismutase	SOD
superoxide dismutase 1	SOD1
toll-like receptor 2	TLR2
Trolox equivalent antioxidant capacity	TEAC or TE
tumor necrosis factor-alpha	TNF-α
xanthohumol	XN
